# Global, regional, and national burden of uterine fibroids in the last 30 years: Estimates from the 1990 to 2019 Global Burden of Disease Study

**DOI:** 10.3389/fmed.2022.1003605

**Published:** 2022-11-07

**Authors:** Lin-Can Cheng, Hao-Yang Li, Qin-Qin Gong, Cheng-Yang Huang, Chao Zhang, Jin-Zhu Yan

**Affiliations:** ^1^Center for Evidence-Based Medicine and Clinical Research, Taihe Hospital, Hubei University of Medicine, Shiyan, China; ^2^Center for Gynecology and Obstetrics, Taihe Hospital, Hubei University of Medicine, Shiyan, China

**Keywords:** uterine fibroids, global burden of disease, disability-adjusted life years, socio-demographic index, age-standardized rate

## Abstract

**Objective:**

To study specific information on trends in incidence, mortality, disability-adjusted life years (DALY) and the corresponding trends among five sociodemographic index regions, 21 regions, and 204 countries for decision-making, which would enable policymakers to distribute limited resources and devise policies more rationally.

**Methods:**

Data on uterine fibroids (UNs) from 1990 to 2019, including incidence, mortality, and DALYs, were obtained from the 2019 Global Burden of Disease Study. An estimated annual percentage change (EAPC) was calculated to assess morbidity, mortality, and DALY trends.

**Results:**

The incident cases of UFs increased from 5,769,658 (95%UI, 7,634,3995–4,274,824) incidences in 1990 to 9,643,336 (95%UI, 7,178,053-12,714,741) incidences in 2017, and the age-standardized incidence rate was steady at 225.67/100,000 persons (95%UI, 167.33–298.87) in 1990 to 241.18/100,000 persons (95%UI, 179,45–318.02) in 2019. The incidence ratio in the high sociodemographic index (SDI) region showed a unimodal distribution, with peaks in 2005. Between 2009 and 2017, the age-standardized death rate of UFs declined globally, especially in low-SDI and low-middle SDI regions. In contrast with 860,619 DALYs (95%UI, 473,067-1,505,289) in 1990, the number of DALYs was 1,378,497 (95%UI, 710,915-2,475,244) in 2019, which had increased significantly, whereas the age-standardized DALY rate decreased expressively with an EAPC of −1.93 (95%CI, from −2.16 to −1.71).

**Conclusion:**

The global burden of UFs increased between 1990 and 2019, and the incidences and DALYs increased prominently worldwide, while the deaths from UFs had no evident growth. Lower SDI regions carried an incremental burden of UFs, while disease reduction was observed in higher SDI regions. It is high time we paid attention to the underprivileged regional quality of life and health protection.

## Introduction

Uterine fibroids (UFs), also known as uterine leiomyoma (UL), fibroma, or leiomyoma, are one of the most prevalent tumors of the female genital tract (1, 2). As for the most common mesenchymal uterine neoplasms, leiomyoma is a kind of benign tumor that develops from mesenchymal or connective tissues. In some ways, the low incidence of UFs has contributed to creating challenges in differentiating them from other types of renal tumors. For example, in imaging modalities, the further definitive diagnosis of a leiomyoma depends on the examination of a specimen (3, 4). According to recent study, the estimated incidence of UFs is between 20 and 77%, and the estimated prevalence is between 40 and 60% among women under the age of 35 and 70% to 80% among women over the age of 50 (4, 5). Besides, black women in the United States are more likely to have this condition (59%), as reported in a previous study (6).

Although UFs are benign tumors, they can cause a number of unpleasant side effects in a woman's life, including irregular and heavy menstrual bleeding (HMB), severe anemia, dysmenorrhea, pelvic pressure and pain, even urinary incontinence, infertility, and early and recurrent miscarriages (7). These risks increase the risk of UFs, such as early menarche, primiparous age, and various reproductive diseases. This raised the likelihood of UFs, which are linked to a high risk of complications in gynecological disorders, pregnancies, and operations (8). Factors including higher parity, late age for first childbirth, and a regular menstrual cycle were associated with reduced risk of UFs. The US has other hazard factors such as age, obesity, low vitamin D, and endogenous and exogenous hormone factors (7).

Furthermore, UFs are one of the most common benign solid tumors in the female reproductive system. However, it can be treated with drugs and surgery. When medication management fails or surgery is the primary treatment option, there are a number of surgical options available, with minimally invasive surgery likely being the preferred choice in most cases. However, a few studies have already been carried out on the global burden of UF incidence, mortality, disability-adjusted life years (DALYs), and estimated annual percentage change (EAPC) in countries and regions around the world. To relieve the burden of this disease on the public health sector and improve women's quality of life, it is crucial to review and analyze the related global trends of UFs.

Global Burden of Disease (GBD) 2019 research included 369 diseases and injured data points in 204 countries and regions (9). Using data from the Global Burden of Disease Study 1990–2019, this study aimed to examine the incidence, mortality, DALYs, and the corresponding trends in UFs by age and socioeconomic status (SES). In this study, we provided specified information on incidence and mortality trends, DALYs, and the corresponding trends in five sociodemographic index regions, 21 regions, and 204 countries for decision-making, which aims to enable policymakers to distribute limited resources and devise policies more rationally.

## Materials and methods

### Data resources

Data on the annual incidence, death, DALYs, and respective age-standardized rate (ASR) of UFs were obtained from the GBD 2019 study (http://ghdx.healthdata.org/gbd-results-tool). Based on national income per capita, the average education level of the population over 15 years old, and the total fertility rate of the population under 25 years old, the SDI was used to classify the 204 countries and regions into one of the five SDI quintiles (high, high-middle, middle, low-medium, and low) as a generalized measure to reflect on the health index in connection with the development of different areas in the GBD study. Dividing the world into 21 GBD regions and 204 countries and territories accurately revealed the differences in incidence, mortality, and DALYs and the corresponding trends in 204 countries and territories from 1990 to 2019.

### Statistical analysis

The age-standardized incidence rate (ASIR), the age-standardized death rate (ASDR), the age-standardized DALY rate, and the corresponding EAPCs were used to assess the trend in UF incidence and mortality. ASRs (per 100,000 population) were calculated using the following formula (10).


$$\begin{array}{l} ASR=\frac{\overset{A}{\underset{i=1}{\sum }}a_{i}w_{i}}{\overset{A}{\underset{i=1}{\sum }}w_{i}}\;\times \;100,000. \end{array}$$

Furthermore, trends in ASIR/ASDR reflected the alternations in human disease or death patterns and risk factors, excluding the influence of the age component on the incidence or mortality of the population, due to which the incidence or mortality had significant variations at different ages.

The DALY model was used to obtain a universally applicable indicator that combined morbidity and mortality. Incidence was measured by assigning disability weights (DWs) to health conditions, where 0 represents the absence of disability, 1 represented a loss “equivalent to death,” and 0 ≤ DW ≤ 1 quantifies the burden that a particular health condition incurs (11, 12). After a condition was assigned its DW, the years lived with disability (YLDs) were calculated as the product of the condition's duration and DW, which explained the incidence. Years of life lost (YLL) is a measure of premature mortality that considers both the frequency of deaths and the age at which they occur. One YLL represented the loss of 1 year of life. YLLs were calculated from the number of deaths multiplied by a global standard life expectancy at the age at which death occurs. Finally, DALYs for a disease or health condition indicate the sum of the YLLs due to premature mortality and the YLDs due to prevalent cases of the disease or health condition in a population (12).

The concept of EAPC was introduced to reflect trends in ASR within specified time intervals and indicated time trends in age-standardized incidence, death, and DALYs rates of UFs: y = α + βx + ε, where y refers to ln (ASR), x represents the calendar year, and β determines the positive or negative trends in ASR. The formula for calculating EAPC is EAPC=100 × (exp(β) – 1), and its 95% confidence interval (CI) was also obtained from the linear model. ρ represents Pearson's correlation coefficient (13).

If the EAPC value and its lower limit of 95%CI are positive, the ASR is considered an upward trend. Conversely, if the EAPC value and its upper boundary of 95%CI are both negative, it is considered a descending trend for the ASR. Otherwise, ASR is considered to be stable. If the uncertainty between the GBD estimation overlaps the EAPC value, the ASR is considered stable, even if the EAPC value is statistically significant (14). In addition, we drew a corresponding scatter plot to observe the relationship between EAPC, ASR, and SDI. All statistical analyses and calculations were performed using R Software (version 4.1.0).

## Results

### Incidence burden of UFs

Globally, the incident cases of URs had an obvious increase from 5,769,658 (95%UI, 7,634,3995–4,274,824) incidences in 1990 to 9,643,336 (95%UI, 7,178,053–12,714,741) incidences in 2019 (Table 1). Contrary to the 67.14% increase in the incidence rate over the past 30 years, the ASIR was steady with 225.67/100,000 persons (95%UI, 167.33–298.87) in 1990 and 241.18/100,000 persons (95%UI, 179,45–318.02) in 2019 (Table 1).

**Table 1:** The incidences and ASIR from 1990 and 2019 and their temporal trends.

	**1990**		**2019**		**1990-2019**
	**Incident cases**	**ASIR per 100,000**	**Incident cases**	**ASIR per 100,000**	**EAPC**
	No.*10^3^(95%UI)	**No. (95%UI)**	No.*10^3^(95%UI)	**No. (95%UI)**	**No. (95% CI)**
Overall	5,769.66(4,274.82–7,634.40)	225.67(167.33–298.87)	9,643.34(7,178.05–12,714.74)	241.18(179.45–318.02)	0.25(0.24–0.27)
**Sociodemographic index**					
High SDI	1,148.29(850.12–1,510.51)	258.00(191.85–338.10)	1,293.91(967.68–1,700.77)	262.37(196.04–344.26)	0.16(−0.02–0.35)
High-middle SDI	1,597.60(1,178.85–2,125.20)	267.44(198.61–353.70)	2,016.16(1,498.31–2,653.76)	254.36(189.78–333.77)	−0.20(−0.23 to −0.17)
Middle SDI	1,541.21(1,141.08–2,060.05)	187.02(138.12–248.69)	2,863.37(2,136.01–3,771.45)	218.56(162.86–287.17)	0.51(0.48–0.53)
Low–middle SDI	1,039.02(772.57–1,383.16)	214.77(159.34–285.23)	2,351.34(1,740.19–3,122.57)	260.21(191.86–346.80)	0.78(0.70–0.85)
Low SDI	440.11(329.44–587.48)	208.77(154.92–278.63)	1,113.19(828.60–1,476.13)	227.28(169.27–302.26)	0.33(0.30–0.36)
**Region**					
Andean Latin America	85.63(63.23–114.85)	492.73(361.31–656.06)	169.71(125.79–223.96)	507.74(376.57–671.75)	0.08(0.07–0.09)
Australasia	9.42(6.78–12.67)	84.54(61.35–113.24)	12.85(9.34–16.98)	85.58(62.69–113.62)	−0.02(−0.05–0.01)
Caribbean	60.76(44.74–81.42)	350.15(256.85–465.24)	87.09(63.66–116.37)	356.44(260.14–476.54)	0.05(0.02–0.07)
Central Asia	119.66(87.65–160.71)	391.83(286.58–520.72)	209.32(152.47–278.30)	417.35(304.39–553.71)	0.23(0.20–0.26)
Central Europe	161.93(118.22–216.77)	247.62(182.28–329.40)	149.86(111.04–196.83)	243.85(183.13–317.37)	−0.03(−0.18–0.12)
Central Latin America	325.48(236.19–429.20)	439.31(319.24–584.78)	590.87(433.54–781.21)	434.88(319.58–574.30)	−0.10(−0.12 to −0.07)
Central Sub-Saharan Africa	52.08(38.47–69.96)	233.91(171.90–309.74)	140.85(104.50–188.29)	248.66(182.90–335.57)	0.20(0.14–0.25)
East Asia	770.63(564.02–1,043.93)	120.39(87.91–162.54)	1,076.26(808.79–1,434.95)	133.11(100.15–176.14)	0.29(0.15–0.43)
Eastern Europe	713.60(524.41–952.84)	583.94(434.35–771.03)	682.55(501.13–912.81)	582.03(431.20–772.04)	0.03(0.01–0.06)
Eastern Sub-Saharan Africa	141.71(105.52–189.44)	187.71(139.90–249.66)	353.77(264.66–473.62)	195.03(144.71–259.91)	0.13(0.12–0.15)
High–income Asia Pacific	241.88(178.69–322.63)	270.76(198.67–363.49)	215.99(163.56–283.42)	286.38(216.32–378.76)	0.22(0.08–0.36)
High-income North America	321.78(241.07–419.71)	201.79(153.55–261.18)	422.91(315.16–545.46)	241.20(180.22–311.98)	0.94(0.55–1.33)
North Africa and the Middle East	172.53(128.63–233.61)	119.05(89.00–159.20)	402.65(296.96–538.71)	124.03(92.02–166.03)	−0.02(−0.07–0.04)
Oceania	3.49(2.58–4.81)	122.09(89.98–165.94)	8.60(6.36–11.66)	131.54(96.93–176.65)	0.23(0.20–0.26)
South Asia	1,102.20(812.97–1,464.25)	234.42(173.22–311.90)	2,717.65(1,998.53–3,611.14)	292.19(215.57–388.80)	0.94(0.82–1.07)
Southeast Asia	299.12(222.22–406.03)	130.23(97.68–174.31)	515.53(386.00–682.27)	140.93(105.92–186.89)	0.26(0.22–0.29)
Southern Latin America	59.77(43.40–79.73)	244.16(177.03–325.74)	93.18(66.94–126.00)	261.08(187.45–351.66)	0.16(0.03–0.29)
Southern Sub-Saharan Africa	122.66(91.12–164.50)	495.38(368.17–651.67)	217.43(159.98–292.07)	499.05(371.04–661.44)	0.05(0.02–0.07)
Tropical Latin America	135.74(102.40–176.14)	179.18(135.66–231.53)	351.71(260.74–461.36)	273.07(202.73–356.19)	1.44(1.33–1.54)
Western Europe	684.45(499.88–915.71)	341.99(250.21–460.14)	710.05(522.06–951.65)	338.62(247.87–453.28)	0.02(−0.05–0.09)
Western Sub-Saharan Africa	185.12(138.11–247.41)	240.93(178.84–319.71)	514.51(381.03–685.54)	254.53(188.46–337.00)	0.11(0.06–0.16)

**Figure 1:**
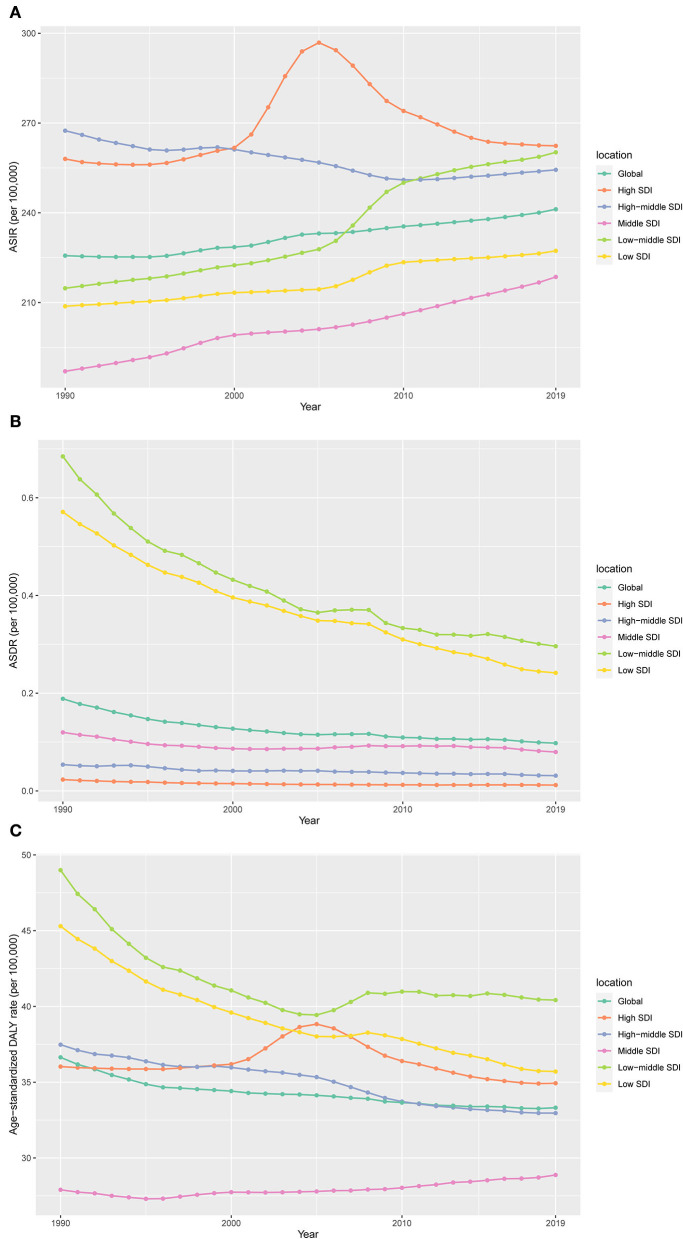
The change trends of age-standardized incidence, death, and DALY rate among different SDI quintiles. **(A)** Age-standardized incidence; **(B)** age-standardized death; **(C)** age-standardized DALYs.

**Figure 2:**
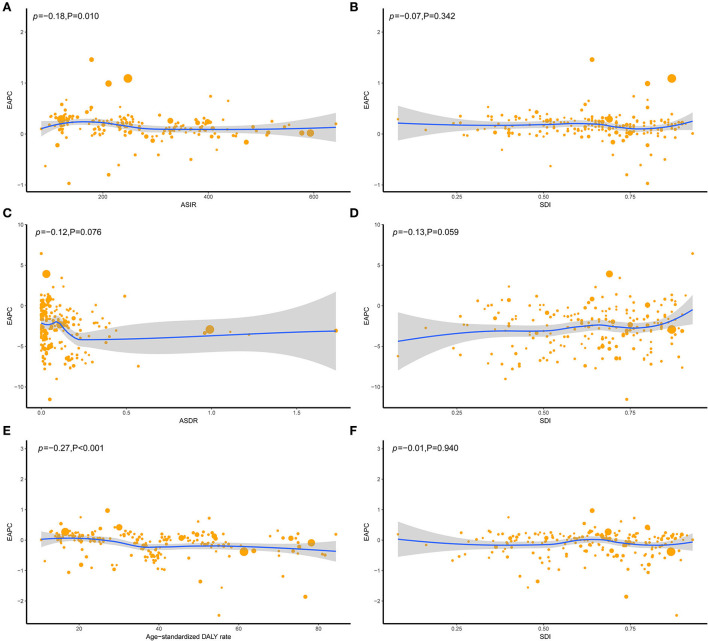
The correlation between EAPC and UFs ASR [incidence **(A)**, death **(C)**, and DALY **(E)**] in 1990 and SDI [incidence **(B)**, death **(D)**, and DALY **(F)**] in 2019. The circles represent countries that were available on SDI data. The size of the circle represents the number of UFs patients, and one circle represents a specific country. The ρ indices Pearson's correlation coefficient and *P*-values were derived from Pearson's correlation analysis. ASR, age-standardized incidence/death/DALYs rate; EAPC, estimated annual percentage change; SDI, socio-demographic index.

According to the SDI level analysis, the ASIR in the high-middle SDI region was on the decline with EAPCs of −0.20 (95%CI, from −0.23 to −0.17). The ASIR of the other four SDI regions was increasing. Moreover, the incidences of different SDI quintiles were mostly on a rising trend, while the high-middle SDI region decreased slowly over the years but consistently above the global level. Strikingly, the high SDI increased sharply after the year 2000, based on a high level for a long time over the world, which peaked in 2005 before declining, which meant that the ratio of incidence in the high-SDI region showed a unimodal distribution, with peaks in 2005. The low-middle SDI had remarkable growth after 2005 and exceeded the global level. The ASIR of low and middle SDI quintiles was always beneath the global level and had no obvious change (Table 1 and Figure 1A). In addition, there were nonsignificant correlations between EAPC and ASIR or SDI (Figures 2A,B).

**Figure 3:**
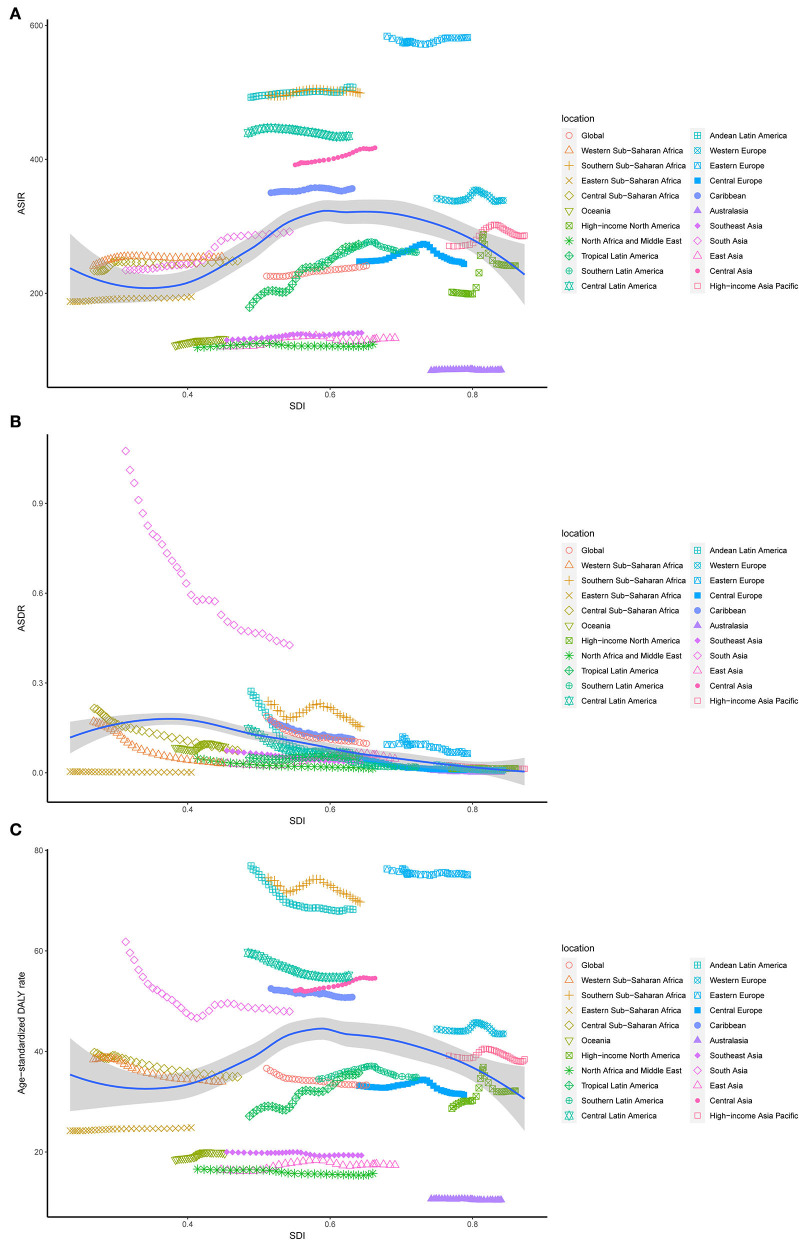
The ASR rates of UFs per 100,000 population among regions based on SDI in 2019. **(A)** Age-standardized incidence; **(B)** age-standardized death; **(C)** age-standardized DALYs.

**Figure 4:**
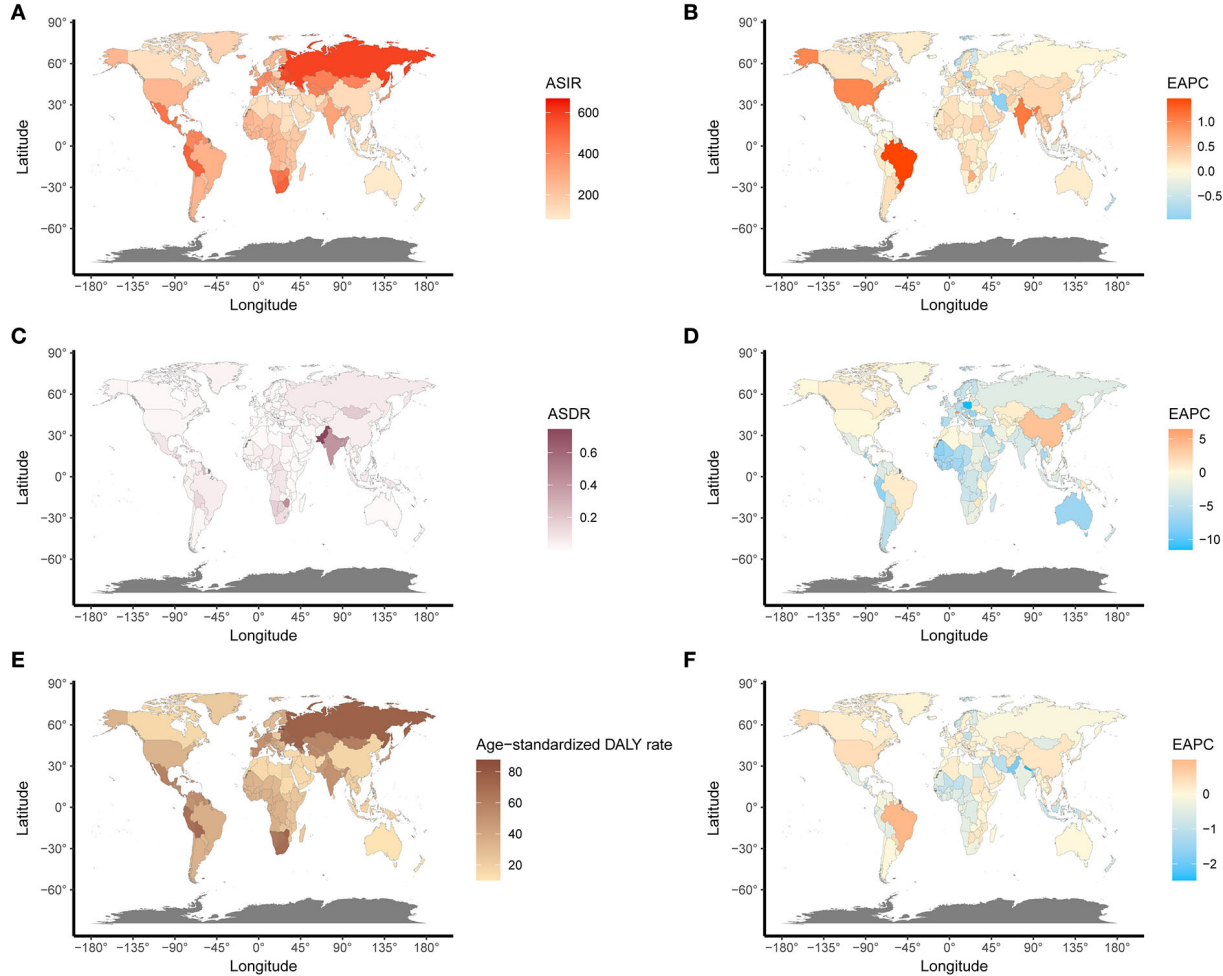
The global disease burden and EAPC of UFs in 204 countries. **(A)** The ASIR of UFs in 2019. **(B)** The EAPC of ASIR. **(C)** The ASDR of UFs in 2019. **(D)** The EAPC of ASDR. **(E)** The age-standardized DALY rate of UFs in 2019. **(F)** The EAPC of age-standardized DALY rate. ASIR, age-standardized incidence rate; ASDR, age-standardized death rate.

**Figure 5:**
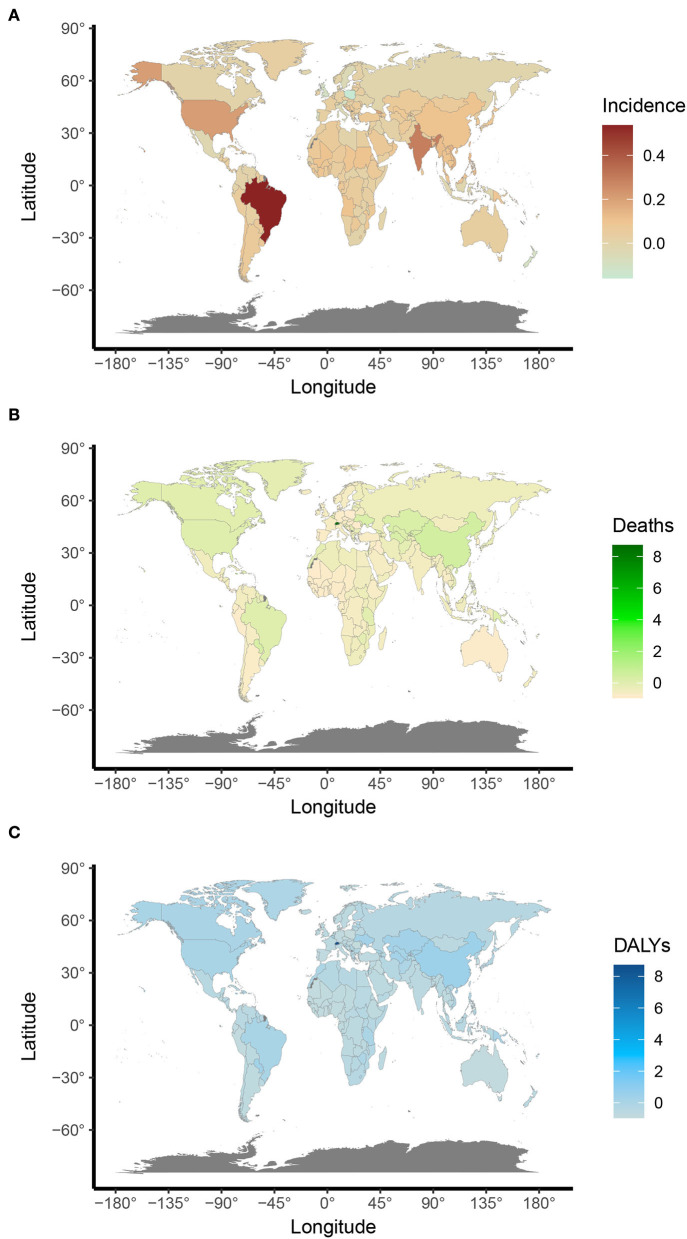
Annual percent changes of age-standardized UFs ASR rates in 204 countries and territories between 1990 and 2019. **(A)** Age-standardized incidence; **(B)** Age-standardized death; and **(C)** Age-standardized DALYs.

On observation from GBD regions and countries level, the incidences increased in most regions, with tropical Latin America leading the way, followed by high-income North America and South Asia, and had an obvious decrease in Central Latin America. Meanwhile, ASIR showed a downward trend in four regions, including Australasia, Central Europe, Central Latin America, North Africa, and the Middle East (Table 1). Among the locations with relatively high SDI, Eastern Europe maintained the highest ASIR, Australasia maintained the lowest ASIR, and other regions approached standard levels. In the middle SDI regions, most countries deviated from the standard level; however, there were no significant differences. The ASIR of the world represents low SDI regions (Figure 3A). Moreover, Ukraine, Russia, and the Republic of Moldova showed the highest ASIR, while New Zealand, Afghanistan, and Australia showed the lowest ASIR in 2019 (Figure 4A). From 1990 to 2019, Brazil had the highest number of reported new cases of UFs, while Poland had the least (Figure 5).

### Deaths burden of UFs

There were slight changes in global annual deaths over the past 30 years, illustrated by 4187.24 (95%UI, 2011.64–6200.21) deaths in 1990 and 4194.17 (95%UI, 2315.10–6479.87) deaths in 2019. However, ASDR significantly decreased, according to the data of an EAPC of −1.93 (95%CI, from −2.16 to −1.71), which dropped from 0.19/100,000 persons (95%UI, 0.09–0.29) in 1990 to 0.10/100,000 persons (95%UI, 0.05–0.15) in 2019 (Table 2).

**Table 2:** The death cases and ASDR in 1990 and 2019 and its temporal trends.

	**1990**		**2019**		**1990–2019**
	**Death cases**	**ASDR per 100,000**	**Death cases**	**ASDR per 100,000**	**EAPC**
	**No. (95% UI)**	**No. (95% UI)**	**No. (95% UI)**	**No. (95% UI)**	**No. (95% CI)**
Overall	4,187.24(2,011.64–6,200.21)	0.19(0.09–0.29)	4,194.17(2,315.10–6,479.87)	0.10(0.05–0.15)	−1.93(−2.16 to −1.71)
**Sociodemographic index**					
High SDI	118.23(87.50–143.84)	0.02(0.02–0.03)	96.17(77.30–133.37)	0.01(0.01–0.02)	−2.04(−2.35 to −1.73)
High–middle SDI	305.67(258.06–509.60)	0.05(0.05–0.09)	315.46(244.07–501.15)	0.03(0.02–0.05)	−1.70(−1.86 to −1.55)
Middle SDI	722.32(405.81–1,128.79)	0.12(0.07–0.19)	1,017.95(474.19–1,432.80)	0.08(0.04–0.11)	−0.76(−1.03 to −0.48)
Low–middle SDI	2,268.43(917.76–3,568.45)	0.68(0.28–1.10)	2,098.55(1,110.71–3,476.49)	0.30(0.15–0.49)	−2.63(−2.87 to −2.39)
Low SDI	771.35(231.16–1,363.14)	0.57(0.17–1.07)	664.66(361.29–1,091.31)	0.24(0.13–0.39)	−2.79(−2.88 to −2.70)
**Region**					
Andean Latin America	39.15(14.22–54.90)	0.27(0.10–0.37)	19.04(12.18–44.86)	0.06(0.04–0.14)	−5.28(−5.99 to −4.57)
Australasia	0.98(0.69–1.28)	0.01(0.01–0.01)	0.28(0.20–0.63)	0.00(0.00–0.00)	−6.35(−7.30 to −5.40)
Caribbean	25.92(17.00–42.30)	0.17(0.11–0.28)	29.26(19.09–46.12)	0.11(0.07–0.18)	−1.46(−1.60 to −1.33)
Central Asia	14.03(9.82–19.28)	0.05(0.04–0.07)	19.53(12.12–24.58)	0.04(0.03–0.05)	−0.32(−0.47 to −0.17)
Central Europe	36.68(22.06–39.94)	0.05(0.03–0.06)	9.35(6.92–15.40)	0.01(0.01–0.02)	−5.78(−6.38 to −5.19)
Central Latin America	78.56(67.05–108.29)	0.15(0.13–0.19)	85.87(65.97–137.08)	0.06(0.05–0.10)	−2.60(−3.13 to −2.06)
Central Sub-Saharan Africa	38.26(4.67–97.55)	0.21(0.03–0.55)	33.33(5.86–67.40)	0.07(0.01–0.15)	−3.60(−3.83 to −3.38)
East Asia	152.56(67.41–538.26)	0.03(0.01–0.12)	492.75(163.27–677.55)	0.05(0.02–0.06)	3.55(2.68–4.43)
Eastern Europe	130.32(99.48–162.27)	0.09(0.07–0.12)	93.33(71.74–131.99)	0.06(0.05–0.09)	−1.86(−2.18 to −1.54)
Eastern Sub-Saharan Africa	2.37(0.46–5.92)	0.00(0.00–0.01)	2.44(0.72–4.59)	0.00(0.00–0.00)	−3.50(−3.67 to −3.33)
High–income Asia Pacific	26.12(21.30–33.45)	0.03(0.02–0.03)	28.68(21.42–39.98)	0.01(0.01–0.02)	−1.93(−2.23 to −1.63)
High–income North America	23.11(15.38–27.13)	0.01(0.01–0.02)	34.23(25.99–48.58)	0.01(0.01–0.02)	0.13(0.00–0.26)
North Africa and the Middle East	49.83(15.13–78.22)	0.05(0.01–0.08)	36.50(11.41–66.93)	0.01(0.00–0.03)	−3.56(−3.87 to −3.26)
Oceania	1.57(0.62–2.85)	0.08(0.04–0.14)	3.85(0.94–7.15)	0.09(0.02–0.15)	0.74(0.47–1.02)
South Asia	3,156.47(1,280.33–4,988.75)	1.07(0.42–1.77)	2,978.91(1,617.77–5,285.66)	0.43(0.23–0.73)	−3.08(−3.24 to −2.92)
Southeast Asia	146.30(32.64–229.22)	0.07(0.01–0.11)	113.00(19.57–187.26)	0.04(0.01–0.06)	−2.61(−2.70 to −2.53)
Southern Latin America	15.56(10.01–18.70)	0.06(0.04–0.08)	7.51(5.67–14.61)	0.02(0.01–0.04)	−4.15(−4.70 to −3.60)
Southern Sub-Saharan Africa	45.69(28.15–94.55)	0.24(0.15–0.50)	55.75(33.19–120.36)	0.15(0.09–0.33)	−0.77(−1.19 to −0.36)
Tropical Latin America	28.39(23.08–38.42)	0.05(0.04–0.07)	72.03(55.00–93.46)	0.05(0.04–0.07)	0.87(0.68–1.06)
Western Europe	72.77(47.90–87.55)	0.03(0.02–0.03)	30.41(23.32–49.11)	0.01(0.01–0.01)	−4.09(−4.59 to −3.58)
Western Sub-Saharan Africa	102.60(11.37–184.80)	0.17(0.02–0.31)	48.12(16.12–78.36)	0.03(0.01–0.05)	−6.47(−6.76 to −6.19)

The ASDR in all the SDI regions was found to decrease in these 30 years, which occupied the low and low-middle SDI quintiles, even though the other quintiles were lower, compared to the global level (Table 2 and Figure 1B). Moreover, nonsignificant correlative relations were observed between the random comparison of EAPC and ASDR or SDI (Figures 2C,D).

Regionally, there was a decline in ASDR in almost all regions except for East Asia, high-income North America, Oceania, and tropical Latin America, in which East Asia increased at best among those regions (Table 2). With the increasing global SDI after 1990, the ASDR of South Asia inversely deviated from the global level, showing an obvious decreasing trend from 1990 to 2019, while the ASDR of Eastern Sub-Saharan Africa remained the lowest for years (Figure 3B). Moreover, it can be concluded that Pakistan, India, Nepal, and Bhutan, adjacent to each other, had the highest ASIR in 2019 (Figure 4).

### DALYs burden of UFs

Globally, the DALYs rose from 860,620 (95%UI, 473,067–1,505,289) in 1990 to 1,378,497 (95%UI, 710,915–2,475,244) in 2019, which is a sizeable increase. However, we witnessed the opposite trend between 1990 and 2019; we found that the age-standardized DALY rate had declined with an EAPC of −1.93 (95%CI, from −2.16 to −1.71), falling from 36.64/100,000 persons (95%UI, 20.15–64.07) in 1990 to 33.32/100,000 persons (95%UI, 17.15–59.92) in 2019 (Table 3).

**Table 3:** The DALYs and age-standardized DALY rate from 1990 and 2019 and its temporal trends.

	**1990**		**2019**		**1990-2019 EAPC No. (95% CI)**
	**DALY** No. *10^3^(95% UI)	**Age-standardized** **DALY Rate** **per 100,000** **No. (95% UI)**	**DALY** No. *10^3^(95% UI)	**Age-standardized** **DALY Rate** **per 100,000** **No. (95% UI)**	
Overall	860.62(473.07–1,505.29)	36.64(20.15–64.07)	1,378.50(710.92–2,475.24)	33.32(17.15–59.92)	−1.93(−2.16 to −1.71)
**Sociodemographic index**					
High SDI	166.28(78.81–307.42)	36.03(17.10–66.86)	207.96(99.06–389.42)	34.93(16.45–65.60)	−2.04(−2.35 to −1.73)
High-middle SDI	218.15(108.17–409.53)	37.48(18.67–70.11)	295.02(142.62–547.54)	32.96(15.94–61.42)	−1.70(−1.86 to −1.55)
Middle SDI	199.78(109.80–349.97)	27.90(15.25–49.20)	393.42(202.33–706.16)	28.88(14.86–51.79)	−0.76(−1.03 to −0.48)
Low-middle SDI	198.47(118.31–318.78)	48.99(29.03–76.83)	338.57(190.78–589.60)	40.42(23.06–70.04)	−2.63(−2.87 to −2.39)
Low SDI	77.48(42.80–126.75)	45.30(24.90–73.06)	142.75(79.06–249.26)	35.70(20.07–62.85)	−2.79(−2.88 to −2.70)
**Region**					
Andean Latin America	11.51(6.40–20.29)	76.91(42.67–136.18)	22.08(10.94–41.23)	68.25(33.82–126.88)	−5.28(−5.99 to −4.57)
Australasia	1.17(0.56–2.21)	10.66(5.13–19.98)	1.80(0.82–3.50)	10.48(4.80–20.33)	−6.35(−7.30 to −5.40)
Caribbean	8.17(4.39–14.35)	52.51(28.17–92.10)	12.97(6.72–23.41)	50.80(26.35–91.71)	−1.46(−1.60 to −1.33)
Central Asia	14.84(7.26–28.29)	52.06(25.34–98.41)	27.01(12.87–51.60)	54.56(26.01–104.46)	−0.32(−0.47 to −0.17)
Central Europe	22.82(11.01–42.55)	33.17(16.09–62.22)	22.18(10.30–41.89)	31.34(14.79–59.46)	−5.78(−6.38 to −5.19)
Central Latin America	36.95(19.25–67.37)	59.61(30.97–107.56)	74.88(36.99–136.66)	55.07(27.24–100.57)	−2.60(−3.13 to −2.06)
Central Sub-Saharan Africa	7.50(3.73–13.07)	39.79(19.90–68.92)	16.70(8.43–30.29)	34.96(17.69–63.25)	−3.60(−3.83 to −3.38)
East Asia	93.49(45.71–172.53)	16.48(8.17–30.24)	164.30(84.29–297.40)	17.39(8.89–31.56)	3.55(2.68–4.43)
Eastern Europe	102.20(50.19–192.79)	76.29(37.47–144.19)	103.95(50.35–196.52)	75.11(35.90–141.98)	−1.86(−2.18 to −1.54)
Eastern Sub-Saharan Africa	15.15(7.20–27.62)	24.23(11.69–44.88)	37.38(17.55–69.20)	24.86(11.71–46.14)	−3.50(−3.67 to −3.33)
High–income Asia Pacific	38.56(18.31–72.41)	39.15(18.55–73.95)	38.98(18.51–72.94)	38.45(18.27–73.44)	−1.93(−2.23 to −1.63)
High–income North America	45.27(21.63–85.29)	28.65(13.67–54.14)	67.48(32.59–124.41)	32.17(15.35–59.74)	0.13(0.00–0.26)
North Africa and the Middle East	20.68(10.59–37.04)	16.60(8.57–29.30)	47.56(22.92–87.07)	15.75(7.57–28.77)	−3.56(−3.87 to −3.26)
Oceania	0.45(0.24–0.77)	18.47(9.86–32.12)	1.13(0.59–2.02)	19.72(10.37–35.13)	0.74(0.47–1.02)
South Asia	235.96(140.45–367.02)	61.80(36.01–93.77)	406.64(234.66–705.53)	47.95(28.01–82.55)	−3.08(−3.24 to −2.92)
Southeast Asia	41.23(22.15–72.58)	20.00(10.71–35.06)	71.68(36.32–128.36)	19.33(9.79–34.61)	−2.61(−2.70 to −2.53)
Southern Latin America	8.37(4.24–15.30)	34.51(17.40–63.24)	13.15(6.16–24.75)	34.90(16.43–65.85)	−4.15(−4.70 to −3.60)
Southern Sub-Saharan Africa	15.58(8.31–27.76)	74.53(39.53–134.75)	27.85(14.37–51.00)	69.72(35.96–127.94)	−0.77(−1.19 to −0.36)
Tropical Latin America	17.94(9.11–32.74)	27.15(13.90–48.78)	47.32(23.56–85.55)	35.73(17.76–64.41)	0.87(0.68–1.06)
Western Europe	98.29(46.15–186.34)	44.47(20.65–83.41)	115.56(53.41–217.76)	43.52(19.99–82.21)	−4.09(−4.59 to −3.58)
Western Sub-Saharan Africa	24.48(12.34–42.71)	38.38(19.65–66.63)	57.85(28.31–105.81)	33.98(16.56–62.16)	−6.47(−6.76 to −6.19)

Similar to the declining ASIR rate, the age-standardized DALY rate for most SDI is falling, with high SDI rising first and then falling (Figures 1A,C). Dominant areas of low and low-middle SDI quintiles existed in the age-standardized DALY rate but not in ASIR. Continuous the highest level of low-middle SDI; however, middle SDI maintained a relatively flat and the lowest level lower than the global level recently. The high-middle SDI is close to the global level and displays a slow downward trend (Figure 1C). A low negative correlation was found between EAPC and DALYs (ρ = −0.27, P < 0.001, Figure 2E), and a nonsignificant correlation was observed between EAPC and SDI (Figure 2F).

Comparing the regions and nations included in the GBD, we found that the age-standardized DALY rate is increasing in East Asia, high-income North America, Oceania, and tropical Latin America, while falling in other regions. Andean Latin America, Australasia, and the Caribbean had the highest age-standardized DALY rates, while Western Sub-Saharan Africa, Western Europe, and tropical Latin America had the lowest (see Table 3). In addition, the relationship between the age-standardized DALY rate of different regions and SDI was similar to that between the ASIR and SDI (Figures 3A,C).

### Age-related incidence of UFs

**Figure 6:**
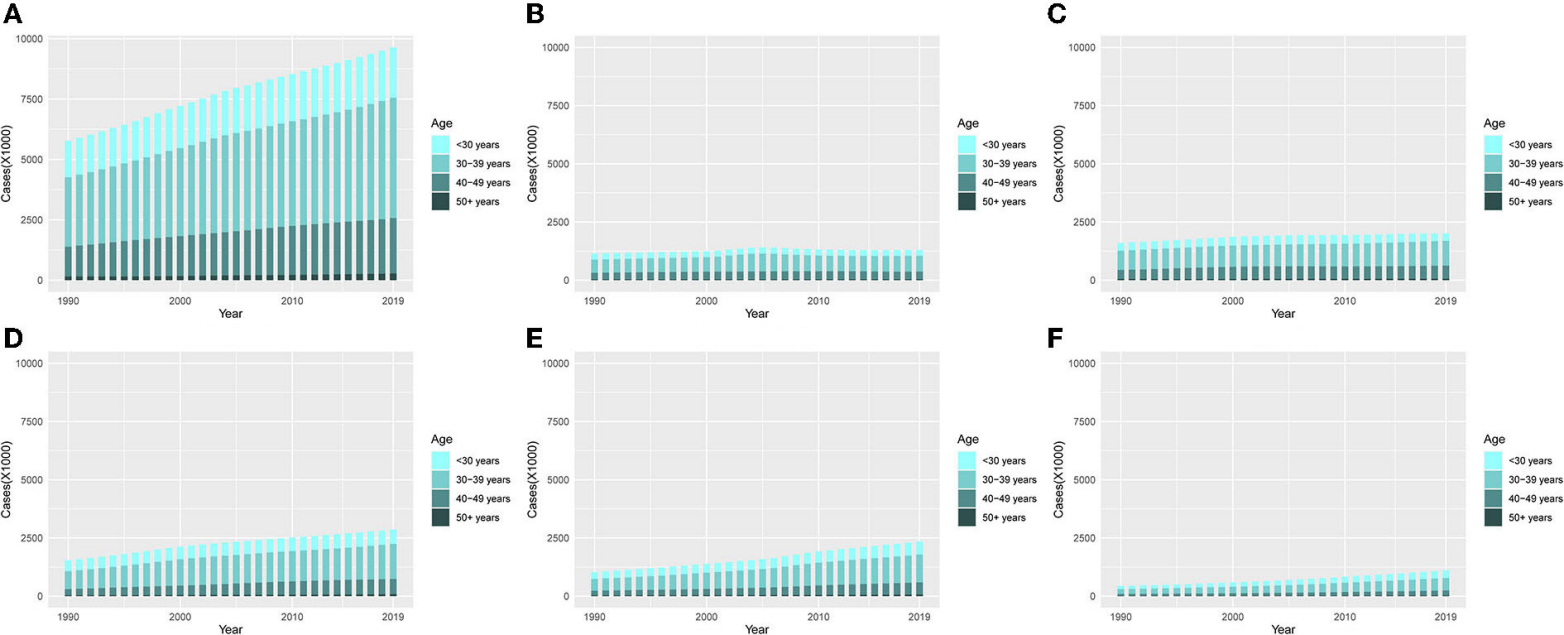
The proportion of the four age groups for incidences of UFs between 1990 and 2019. **(A)** Global. **(B)** High SDI. **(C)** High-middle SDI. **(D)** Middle SDI. **(E)** Low-middle. **(F)** Low SDI.

On the whole, it can be seen from Figure 6A that the incidence of UFs in the 30–39 year age group accounted for the highest proportion among the other four age groups (< 30 years, 30–39 years, 40–49 years, and 50+ years), and cases in all the age groups were on a rising trend with the increase of years.

From a single observation of different SDI regions, it can be seen that the ASIR in the middle SDI, low-middle SDI, and low SDI quintiles showed the most obvious trend of change, among which the 30–39-year age group had a clear escalation (Figures 6B–F).

**Figure 7:**
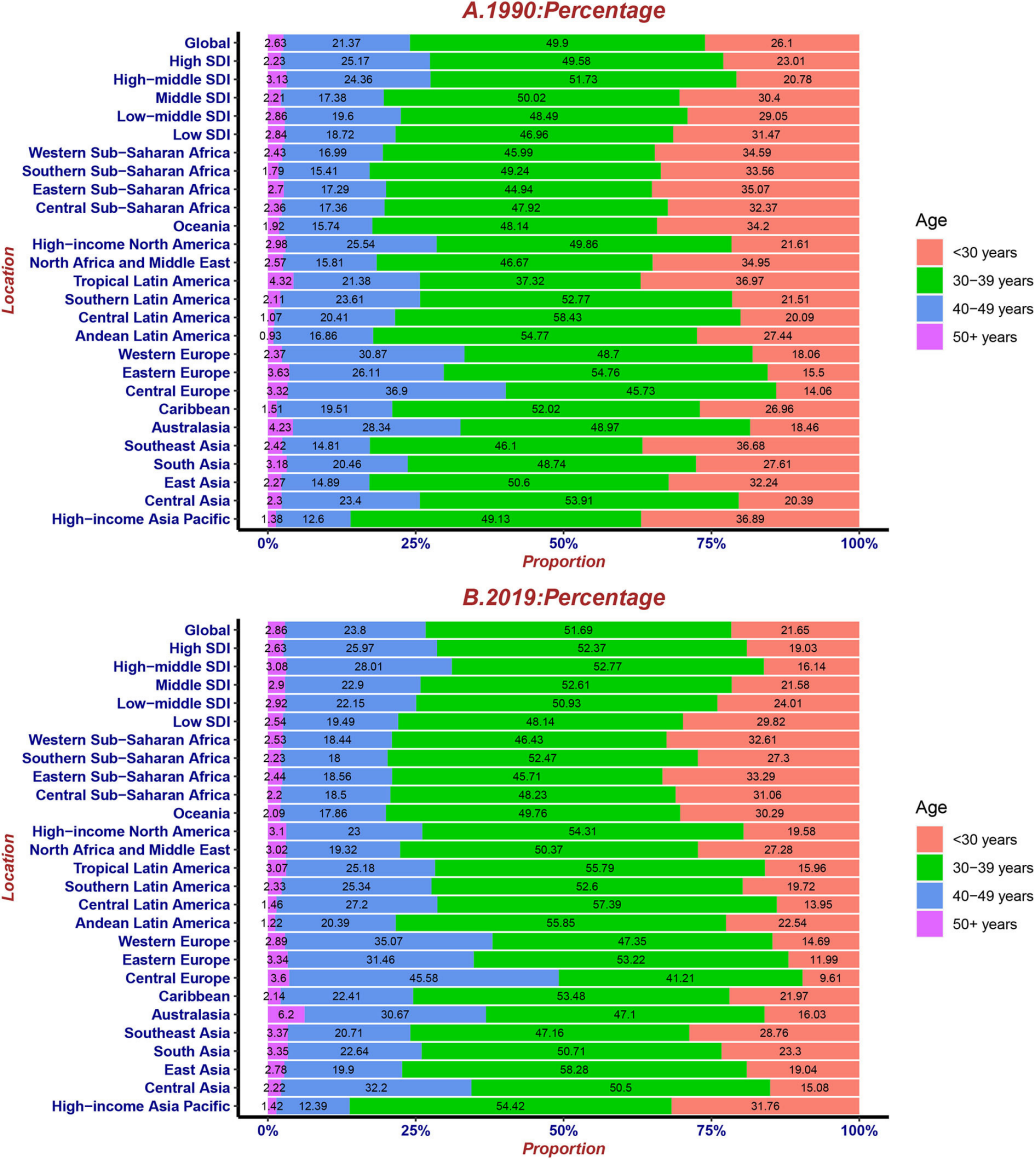
The proportion of four age groups for UFs incident cases globally and by region, contrasted in 1990 **(A)** and 2019 **(B)**.

Overall, the proportion of cases in the four age groups did not change typically during the last 30 years. The number of cases in the < 30 years and 30–39 years groups declined, while those in the 40–49 years and >50 years groups were on the rise. In particular, the percentage of cases in < 30 years slumped in tropical Latin America. Furthermore, compared with other regions, 40–49 years had the largest proportion of cases, while < 30 years had the lowest proportion of cases in Central Europe in 1990 and 2019. Besides, >50 years had the largest percentage in Australasia (Figure 7).

## Discussion

This study used data from GBD 2019 to show how the prevalence of UFs has changed over the past 30 years. UFs are the most prevalent pelvic tumors in women of reproductive age, impacting over 70% of women overall and disproportionately harming women of color (5, 15, 16). In recent years, there have been no global epidemiological studies on UFs. Since UFs are benign tumors with a low mortality rate compared with other cancer types, there are no relevant GBD articles to investigate the underlying mechanism behind UFs. Our goal in documenting the global burden of UFs is to provide decision-makers with comprehensive, representative, and instructive evidence.

A total of 3,873,678 new cases were reported in 2019, with a growth rate of 67.14% compared to the global incident cases of UFs from 1990 to 2019, but changes in ASIR (6.87% increase) were less prominent. The deaths and the DALYs of UFs worldwide increased by 0.17 and 60.18% between 1990 and 2019, respectively. Although the growth of the DALYs of UFs changed noticeably, the age-standardized DALY rate decreased significantly with an EAPC of −1.93. In the low-middle SDI and the low SDI regions, the ASDR and the age-standardized DALY rate were relatively higher, and their trends were similar from 1990 to 2019. It was worth noting that the high-SDI region had the lowest ASDR, while the age-standardized DALY rate showed increasing firstly and then decreasing trends, and its value was not low. According to a systematic analysis of the global burden of disease study, the changing age structure, population growth, and changing age-specific incidence rates can be influencing factors for changing cancer incident cases (17). The reasons for the rising cancer rates varied greatly across SDIs. Population growth was regarded as the main contributor to the increase in all cancer incidences, especially in the low SDI quintile. In low-middle SDI countries, aging and changes in incidence rates played their roles equally (each 12%). The rising incidence in high-middle and high-SDI countries was attributable to the aging population (17).

In particular, the prevalence of UFs was higher in high-SDI and high-middle SDI regions and relatively lower in low and low-middle SDI regions. However, the deaths and the age-standardized DALY rate of UFs were lower in high-SDI regions than in low-SDI regions. A possible explanation for this observation is that greater medical and diagnostic levels correspond to higher SDI values. People with a better quality of life would pay more attention to maintaining their health, eventually resulting in the highest incidences but lowest deaths in the high SDI quintile. Except for the high-middle SDI quintile, the increased incidence of UFs was observed in the rest of the areas, with the greatest increase in the low-middle areas but the least increase in the high-SDI countries.

Meanwhile, the incidence of UFs was associated with regions. For instance, the highest ASIR was observed in Ukraine, Russia, and the Republic of Moldova, while New Zealand, Afghanistan, and Australia showed the lowest ASIR in 2019. Different countries also presented various incidences of UFs despite being in the same SDI regions. Among the locations with relatively high SDI, Eastern Europe had the highest ASIR. In contrast, Australia maintained the lowest ASIR, and other regions approached standardized levels while considering the race factor. Race was the most important and frequently reported risk factor contributing to UFs (18), which could be proven by the disproportionate incidences in different regions and countries nationwide. Some studies showed that there was a positive adjustment correlation between UFs' risks and self-described African American ethnicity (19).

Moreover, increasing age was also identified as a potential risk factor attributable to UFs, especially among women at the premenopausal stage and those aged at least 40 years (19–21). In our study, the incidence ratio increased in the 40–49-year age groups in the middle-SDI and low-middle-SDI regions, but it was relatively flat in the other three SDI regions. The incidence of UFs in the elder age groups was increasing globally, which may be attributed to the aging population. The number of incidences worldwide increased significantly in the 30–39-year age group. Except for the high-SDI region, the number of 30–39-year age group incidences in the other four SDI regions showed an upward trend.

The proportion of incidences and deaths in young adults decreased in all its cases, while the proportion of incidences and deaths in the elderly increased between 1990 and 2019. This may be because these tumors were not detected in girls before puberty. Due to the tiny differences in the biochemical pathways, young girls did not exhibit typical uterine fibroid biological characteristics. At the onset of menopause, UFs began to atrophy, and sex hormones were reduced. Notably, the use of hormone replacement therapy can cause these lesions to grow and may cause the initial clinical symptoms of UFs to manifest (18).

With the rapid development of science and technology along with the deterioration of the ecological environment in recent years, life's risk factors have gradually increased, which is closely related to the incidence of UFs. Some adverse environmental exposures, such as air pollution and alcohol consumption, were regarded as risk factors for UFs (18). Air pollution is one of the leading causes of death. Air pollution caused by PM 2.5 causes menstrual irregularity, infertility, endometriosis, and long-term exposure to PM of 2.5 causes clinical symptoms of UFs to manifest (22). Furthermore, heavy alcohol consumption was one significant risk factor for UFs (23). A study revealed that drinking at least 20 glasses of alcohol daily led to an increased risk of UFs (24). Another cross-section study indicated that the consumption of alcohol was higher in women with UFs than in those without (25). Alcohol use changed the cytokines and growth factors that played a vital role in uterine fibroid pathogenesis.

Moreover, alcohol-induced DNA damage might be a significant contributor (18). These findings were consistent with the ASIR, ASDR, and age-standardized DALY rates. Furthermore, obesity was also identified as a potential risk factor for its development. Consistently, the incidence of UFs was highest in the high SDI and the high-middle SDI quintiles. Also, with the growth of people's quality of life in recent years, the incidence of UFs in low-middle SDI regions also increased significantly. In light of Hyuna Sung's study (26), the most obvious obesity increase occurred among women in the Middle East, Central Asia, and North Africa, which can be attributed to the global food system changes producing high-calorie and low-nutrient foods. Moreover, obesity is associated with decreased physical activity. (27). The risk of developing UFs rises with each additional kilogram of excess weight (28, 29), and it was found to be more prevalent in African Americans. The U.S.'s other racial and ethnic groups had the highest rates of obesity. (30). However, the prevalence of obesity was not always correlated to prosperity; some Asian Pacific countries were accustomed to maintaining their conventional low-calorie dietary habits, and the prevalence of obesity was lowest in those who engaged in only the most basic forms of physical activity (31). The prevalence of obesity in some low-income countries, such as the Pacific Island nations and Egypt, was considerably high (32).

Uterine leiomyomas or fibromas were a common cause of infertility and a major indication for hysterectomy (33). Although it mainly affects older women from an epidemiological point of view, it can also affect young patients who want children and have not yet completed their birth plans (34). Therefore, fertility preservation therapy (FST) was needed to treat uterine fibroids to maintain fertility. Standard treatments for endometrial cancer (EC), including total hysterectomy and bilateral salpingo-oophorectomy (TH-BSO), had good results in terms of survival rate but also led to permanent loss of fertility. Fertility-sparing methods included hormonal therapy with oral progestins and/or levonorgestrel-releasing IUD (LNG-IUD), hysteroscopic surgery, and combined use of metformin for evaluation (35).

Traditional surgical treatments for symptomatic UFs include myomectomy and hysterectomy. However, for this common gynecological disease, minimally invasive surgery and image-guided ablation technology gradually occupied the dominant position in the treatment prospect, such as uterine artery embolization (UAE) with definitive efficacy and high safety and high intensity focused ultrasound (HIFU) guided by non-invasive ultrasound or magnetic resonance imaging (MRI)(36, 37). Compared with traditional laparoscopic myomectomy, HIFU guided by ultrasound can improve patients' quality of life more effectively, and it is helpful for the recovery and prognosis of hysteromyoma after treatment (38). However, it is necessary to guard against complications and adverse events (AEs) when considering therapeutic use (39).

Policymakers and governments require country-specific information on the global burden of different diseases to adjust their national benchmarks, implement relevant measures to reduce disease incidence, and allocate limited resources in their healthcare systems. For example, failure to promptly detect new cases of UFs was a concern for countries in the low-SDI region. Considering that the existing data in many countries are rarely accurate or missing, GBD research results can be used to study the trends of different diseases in their respective locations.

GBD studies provided a high-quality assessment of the global disease burden; however, they are still several limitations. An inevitable restriction is the uncertainty of GBD estimation, where the actual data on the disease burden is unavailable. The discrepancies in the data collected by different data extraction methods and the accuracy of different studies are also unavoidable in this analysis mode. The fluctuations in the incidence and mortality may be partially reflected in the detection deviation associated with the adjustment in the screening scheme rather than the actual change in the age rate at a particular age. In addition, owing to GBD 2019 data, there was a lack of risk factors for UFs; this article did not extract relevant precision data to verify the data provided by relevant literature, but this article still has a significant reference value.

## Conclusions

The global burden of UFs kept mounting between 1990 and 2019, and the incidences and DALYs increased significantly worldwide, while the deaths of UFs had no obvious growth. In recent years, lower SDI regions carried an incremental burden of UFs, while disease reduction was observed in higher SDI regions, which may further aggravate the gap between the rich and the poor in the years to come. Since underdeveloped countries were affected to a greater degree by increased health burden, the ASDR of UFs in the low and low-middle-SDI regions was the highest, which meant that it is time to pay attention to the underprivileged regional quality of life and health protection. The incidence of UFs was largely prevalent in middle-aged and older women; it was rare in young women. Screening for gynecological diseases among the middle-aged and elderly should be increased, and measures should be taken to treat diseases as early as possible. Also, measures against attributable risk factors should be taken to reduce the UF burden. Considering these discrepancies, health authorities and policymakers should make the best use of limited resources and develop prevention and control policies that rationalize UFs in their situations.

## Data availability statement

To download the data used in these analyses, please visit the Global Health Data Exchange GBD 2019 data-input sources tool at http://ghdx.healthdata.org/gbd-2019/data-input-sources.

## Author contributions

J-ZY designed the research. C-YH, H-YL, and L-CC collected the data and verified the accuracy of the data. H-YL and L-CC verified the accuracy of the data. CZ, Q-QG, C-YH, and H-YL contributed to data interpretation. H-YL, L-CC, J-ZY, and C-YH performed the statistical analysis and visualization. L-CC and H-YL wrote the manuscript. All authors read, critically reviewed, and approved the final manuscript.

## Conflict of interest

The authors declare that the research was conducted in the absence of any commercial or financial relationships that could be construed as a potential conflict of interest.

## Publisher's note

All claims expressed in this article are solely those of the authors and do not necessarily represent those of their affiliated organizations, or those of the publisher, the editors and the reviewers. Any product that may be evaluated in this article, or claim that may be made by its manufacturer, is not guaranteed or endorsed by the publisher.
